# The Effect of a Dodecahedron-Shaped Structure on the Properties of an Enzyme

**DOI:** 10.3390/jfb13040166

**Published:** 2022-09-28

**Authors:** Yuri D. Ivanov, Vadim Y. Tatur, Ivan D. Shumov, Andrey F. Kozlov, Anastasia A. Valueva, Irina A. Ivanova, Maria O. Ershova, Nina D. Ivanova, Igor N. Stepanov, Andrei A. Lukyanitsa, Vadim S. Ziborov

**Affiliations:** 1 Institute of Biomedical Chemistry, Pogodinskaya Str., 10 Build. 8, 119121 Moscow, Russia; 2 Joint Institute for High Temperatures of the Russian Academy of Sciences, 125412 Moscow, Russia; 3 Foundation of Perspective Technologies and Novations, 115682 Moscow, Russia; 4 Moscow State Academy of Veterinary Medicine and Biotechnology Named after Skryabin, 109472 Moscow, Russia; 5 Faculty of Computational Mathematics and Cybernetics, Moscow State University, 119991 Moscow, Russia

**Keywords:** atomic force microscopy, dodecahedral structure, electromagnetic field, protein aggregation, enzyme adsorption, biosensor

## Abstract

In this research, the influence of a dodecahedron-shaped structure on the adsorption behavior of a horseradish peroxidase (HRP) enzyme glycoprotein onto mica substrates was studied. In the experiments, samples of an aqueous HRP solution were incubated at various distances (0.03 m, 2 m, 5 m, and control at 20 m) from the dodecahedron surface. After the incubation, the direct adsorption of HRP onto mica substrates immersed in the solutions was performed, and the mica-adsorbed HRP particles were visualized via atomic force microscopy (AFM). The effect of the increased HRP aggregation was only observed after the incubation of the enzyme solution at the 2 m distance from the dodecahedron. In addition, with respect to the control sample, spectrophotometric measurements revealed no change in the HRP enzymatic activity after the incubation at any of the distances studied. The results reported herein can be of use in the modeling of the possible influences of various spatial structures on biological objects in the development of biosensors and other electronic equipment.

## 1. Introduction

Electromagnetic radiation is becoming widely employed in modern life, leading to increasing levels of electromagnetic background. This is why studying the influence of electromagnetic radiation on biological objects has become an actual task of modern science. While the levels of ionizing radiation are tightly controlled, radiofrequency radiation (which is widely employed in everyday life) is also able to influence biological objects [1].

The effect of radiofrequency radiation on biological objects depends on its power: while high-power radiation produces well-distinguishable thermal effects [2,3], low-power radiation can lead to various nonthermal effects [2,4]. The use of high-power (600 to 1200 W) microwave (2.4 GHz) electromagnetic radiation for the disinfection of objects contaminated with extremely dangerous infectious microorganisms was reported [5]. Furthermore, low-power (~0.1 µW) radiofrequency (1 GHz) radiation can influence nonspecific antiviral protection systems in animals and humans, modulating the expression of receptors for pathogenicity factors in blood cells [6]. External 1.2 to 1.3 GHz electromagnetic fields, emitted by radars, were reported to affect the levels of erythrocytes and leukocytes in the blood even at low (10 µW to 20 mW) radiation levels [7]. Furthermore, near-background levels of radiofrequency radiation can be used for the correction of the functional state of whole blood cells [8]. Nonthermal microwave radiation with picosecond rise times can be employed in tumor treatment [9]. Moreover, radiofrequency radiation was also reported to influence biological objects at the molecular level, affecting antibody affinity [10] and enzymatic activity [11]. Low-power electromagnetic radiation was reported to influence the activity of enzymes, including horseradish peroxidase (HRP) and other peroxidases [12,13]. Specifically, Lopes et al. [14] demonstrated that 30 min incubation of HRP in a microwave reactor at 60 W radiation power and 60 °C temperature causes a very significant (about 80%) decrease in its enzymatic activity. Interestingly, Yao et al. [15] demonstrated that radiofrequency (27.12 MHz, 6 kW) heating has quite opposite effects on the HRP enzymatic activity depending on the treatment temperature: while the treatment at 50 °C induces a slight (by 5 to 14%) increase in the enzymatic activity, at higher (70 °C and 90 °C) temperatures, this activity is suppressed by 7% to about 50%. Furthermore, Fortune et al. [16] emphasized that the exposure of HRP to a radiation frequency of 13.56 MHz, 915 MHz, or 2.45 GHz does not cause any nonthermal damage to the enzyme, with its enzymatic activity remaining virtually unchanged even after 24 h irradiation at 50 °C.

As regards the electromagnetic and magnetic fields of lower frequency, Caliga et al. [17] reported a nearly twofold decrease in the enzymatic activity of HRP after its exposure to a 50 Hz, 2.7 mT electromagnetic field; however, its enzymatic activity was unaffected by a 100 Hz, 5.5 mT field. Wasak et al. [18] demonstrated that the effect of a rotating magnetic field of an extremely low (1 to 50 Hz) frequency on HRP’s enzymatic activity depends on the parameters of the field, which can either enhance or suppress the enzymatic activity. Emamdadi et al. [19] reported a 30% increase in the enzymatic activity of HRP after its 10 min exposure to a static magnetic field. These authors explained the modulation of the enzyme’s activity by the interaction of the magnetic field with the enzyme structure [19]. As regards other enzymes, Latorre et al. [3] showed that even a short-time exposure (30 s) of red beet peroxidase and polyphenoloxidase to a 2450 GHz, 450 W microwave radiation leads to a very significant (15-fold and 100-fold for red beet peroxidase and polyphenoloxidase, respectively) suppression of their enzymatic activity.

It should be emphasized that geometric bodies of various shapes are able to concentrate background electromagnetic radiation at certain points in space [20]. Balezin et al. theoretically showed the ability of pyramidal structures to alter the spatial distribution of the external background electromagnetic radiation, concentrating the electromagnetic energy near the base of the pyramid [20]. Such a concentration of electromagnetic energy by pyramidal structures was recently found to induce changes in the properties of an enzyme: through AFM, changes in the adsorption properties of HRP after its incubation in certain points near a pyramidal structure were revealed [21].

Macromolecular adsorption can be affected by various external factors, including electromagnetic fields. Under the influence of electromagnetic fields, macromolecules can adsorb onto solid substrates in the form of self-assembled monolayers [22]. Under the action of alternating electromagnetic fields, horseradish peroxidase was shown to adsorb in various forms (for instance, as extended thread-like structures), which depend on the field’s parameters [22]. Moreover, it should be emphasized that in biosensors, biological macromolecules are often adsorbed onto solid substrates, which bear an additional functional layer on their surface; the latter can be represented by a self-assembled monolayer [23,24]. In this case, electromagnetic fields can not only have direct effects on the adsorbate but also can indirectly influence macromolecular adsorption by inducing transitions in the substrate’s functional layer conformation; the latter leads to a change in the polarity of the substrate surface [24].

The list of shapes of spatial structures that are able to cause the spatial redistribution of electromagnetic fields is not limited to pyramidal ones: the incubation of an enzyme solution near objects of spherical shape can also induce changes in the enzyme’s properties [25]. The alterations in the background electromagnetic field topography in the vicinity of the structures of certain shapes occur due to the reflection and refraction of electromagnetic radiation. Specifically, background electromagnetic radiation is reflected from and/or refracted on these structures’ elements, the dimensions of which are of the order of the radiation wavelength [20]. In practice, the phenomenon of changes in the electromagnetic field topography near spatial structures was used in the construction of anechoic chambers, in which pyramidal structures were employed [26].

Currently, a growing interest is directed at the scientific and technical applications of dodecahedral structures. A dodecahedron is a regular polyhedron, the faces of which represent regular pentagons. This structure is represented in nature by various viral particles (such as poliomyelitis virus [27] and adenovirus [28]). The Circorrhegma dodecahedra microorganism has also a near-dodecahedral shape [29].

As regards the application in practice, the use of dodecahedral structures in the development of microwave absorbers was reported [30]. Moreover, particles with dodecahedral shapes are known to be employed in the construction of various biosensors, including enzyme-based ones [31,32,33,34]. Furthermore, dodecahedral structures are widely employed in the construction of omnidirectional sound sources, which are widely employed in acoustic measurements [35,36,37,38]. These facts emphasize the importance of studying the possible effects of the incubation of enzymes in the vicinity of dodecahedron-shaped structures on their properties.

## 2. Materials and Methods

### 2.1. Chemicals and Enzyme

The peroxidase solution used in the experiments was prepared from a commercial preparation purchased from Sigma (Cat. #6782). The 2,2′-azino-bis(3-ethylbenzothiazoline-6-sulfonate) (ABTS) substrate was purchased from Sigma. Disodium hydrogen orthophosphate (Na_2_HPO_4_), citric acid and hydrogen peroxide (H_2_O_2_) were all of an analytical or higher-purity grade and were purchased from Reakhim (Moscow, Russia). Dulbecco’s modified phosphate-buffered saline (PBSD) was prepared by dissolving a salt mixture, commercially available from Pierce, in ultrapure water. All the solutions used in our experiments were prepared using deionized ultrapure water (with 18.2 MΩ×cm resistivity), obtained with a Simplicity UV system (Millipore, Molsheim, France).

### 2.2. Experimental Setup

**Figure 1:**
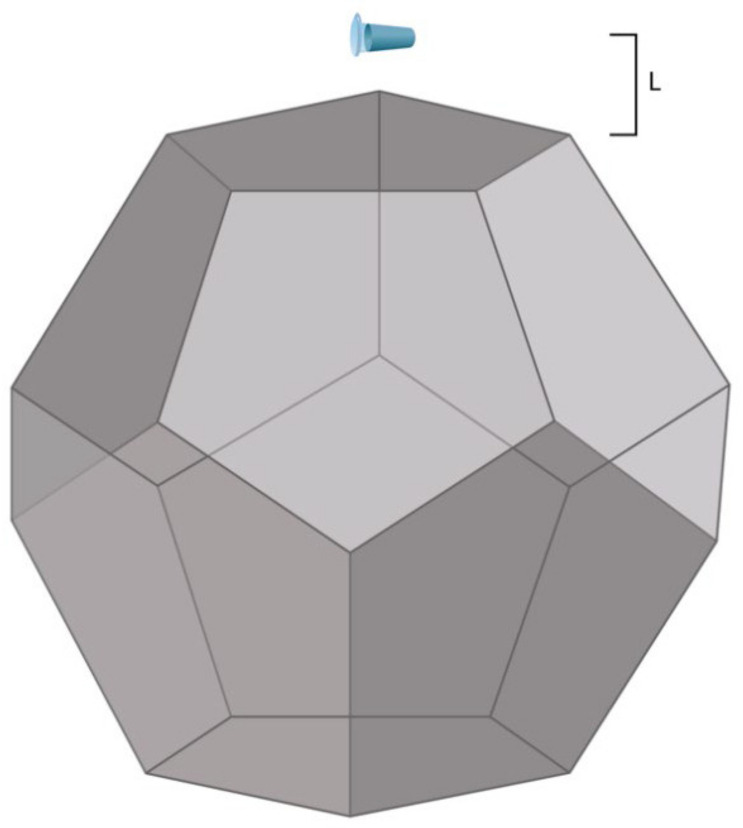
Experimental setup. The distance *L* between the dodecahedron and the tube containing 1 mL of 10^−7^ M HRP solution in 2 mM PBSD (pH 7.4) was either 0.03 m, 2 m, 5 m, or 20 m (the latter value corresponded to the control experiment).

In order to investigate the influence of a dodecahedral structure on the enzyme solution, we used an experimental setup, which is schematically shown in Figure 1.

The edge (*E*) of the dodecahedron was 0.23 m, which is directly proportional to the base side of the pyramid studied by Balezin et al. (230 m [20]). Accordingly, its surface area (*A*) was 1.0922 m^2^. The dodecahedron material was metalized textolite [21]. The laboratory room, wherein the experimental setup was placed, was not electromagnetically shielded. The background electromagnetic field was only induced by common laboratory equipment and was not intentionally generated. The electromagnetic field intensity was not measured.

Briefly, 1 mL of a sample solution, containing 10^−7^ M HRP in 2 mM PBSD (pH 7.4), was placed into an Eppendorf-type test tube (of 1.7 mL nominal volume; SSIBio, Lodi, CA, USA). The tube was incubated at room temperature (25 °C) for 40 min at one of the following distances (*L*) from the center of the dodecahedron:

1. *L*_1_ = 20 m (control experiment);
2. *L*_2_ = 0.03 m (in the vicinity of the dodecahedron; *L*_2_ ≈ 0.1 × *E*);
3. *L*_3_ = 2 m (middle distance; *L*_3_ ≈ 10 × *E*);
4. *L*_4_ = 5 m (long distance; *L*_4_ ≈ 15 × *E*).

**Figure 2:**
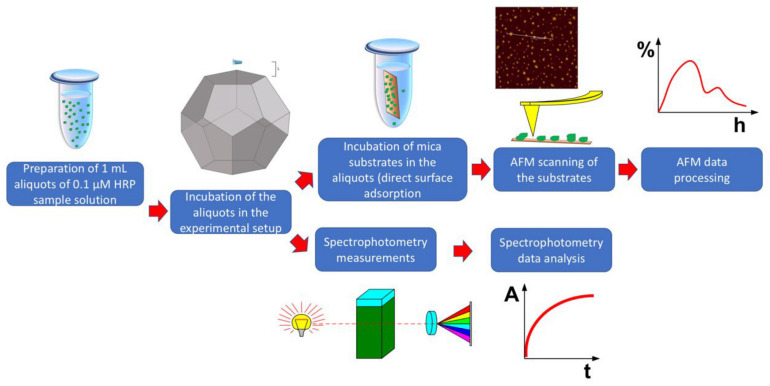
Schematic diagram of the experimental workflow.

These incubation distances were chosen with regard to the edge size (*E*), as specified above. After a 40 min incubation process at any of the above distances from the dodecahedron, the sample solutions were studied via AFM and spectrophotometry. Figure 2 displays a schematic diagram of the experimental workflow.

### 2.3. Direct Surface Adsorption of HRP onto Mica

The AFM analysis technique was based on the method of direct surface adsorption, developed by Kiselyova et al. [39]. Specifically, HRP was directly adsorbed onto the surface of a mica (SPI, USA) substrate incubated in the enzyme solution to be analyzed. The volume of the HRP sample solution was 1 mL, while its concentration was 10^−7^ M. At higher concentrations, HRP tends to form continuous layers on mica substrates, thus hindering the visualization of distinct single molecules [40] and/or low-order aggregates. Ignatenko et al. [41] also reported a considerable aggregation of HRP at micromolar concentrations. Accordingly, we performed our experiments with 10^−7^ M HRP solutions in order to avoid a concentration-induced aggregation of the enzyme. The 7 × 15 mm mica substrate was incubated in the sample solution for 10 min in an Eppendorf Thermomixer Comfort shaker at 600 rpm and room temperature. After such an incubation process, the substrate was rinsed with ultrapure water and dried in air.

### 2.4. Atomic Force Microscopy

AFM scanning was performed following the technique described in our previous papers [13,21,25,40,42,43,44,45]. AFM images were obtained in an intermittent contact mode in air employing a Titanium multimode atomic force microscope (NT-MDT, Zelenograd, Russia) equipped with NSG10 cantilevers (TipsNano, Zelenograd, Russia; 47 to 150 kHz resonant frequency, 0.35 to 6.1 N/m force constant; the microscope pertains to the equipment of the “Human Proteome” Core Facility of the Institute of Biomedical Chemistry, supported by Ministry of Education and Science of Russian Federation, Agreement 14.621.21.0017, unique project ID: RFMEFI62117X0017). The height calibration of the microscope was performed using a TGZ1 calibration grating with a step height of 21.4 ± 1.5 nm (NT-MDT, Zelenograd, Russia). The number of frames obtained for each substrate was ≥10. Similar to [46], the relative density of the distribution of the imaged objects with height *ρ*(*h*) was calculated as follows:

*ρ*(*h*) = (*N_h_*/*N*) × 100%, (1)

where *N_h_* is the number of imaged proteins with height *h*, and *N* is the total number of imaged distinct particles [46].

The relative density of the distribution of the imaged objects with the area was calculated as

*ρ*(*s*) = (*N_s_*/*N*) × 100% (2)

where *N_s_* is the number of imaged objects with area *s*, and *N* is the total number of the imaged objects.

In order to ensure the reliability of the AFM measurements, we performed preliminary experiments with the use of a protein-free buffer instead of an HRP solution. In the preliminary experiments, we did not observe any objects with heights > 0.5 nm. The AFM operation (including the obtaining AFM images), their treatment (flattening correction, etc.), and the export of the obtained data to the ASCII format were performed with a standard NOVA Px software program (NT-MDT, Zelenograd, Russia), supplied with the microscope. The number of particles visualized in the AFM images and the distributions of the particles with the height and area variables were automatically calculated with a specialized AFM data processing software program developed at the Institute of Biomedical Chemistry in collaboration with the Foundation of Perspective Technologies and Novations.

### 2.5. Spectrophotometry

The HRP activity was estimated according to the technique described in detail by Sanders et al. by using ABTS as a substrate in a phosphate–citrate buffer [47] at pH 5.0 [47,48]. The rate of change in absorbance at 405 nm was measured with an Agilent 8453 UV–visible spectrophotometer (Agilent Technologies Deutschland GmbH, Waldbronn, Germany). Then, 2.96 mL of 0.3 mM ABTS solution in the phosphate–citrate buffer (51 mM Na_2_HPO_4_, 24 mM citric acid, pH 5.0) and 30 µL of 10^−7^ M HRP solution were pipetted into a 3 mL quartz spectrophotometric cell of 1 cm pathlength (Agilent Technologies Deutschland GmbH, Waldbronn, Germany), so that the final concentration of the enzyme in the cell was 10^−9^ M. Then, 8.5 mL of 3% (*w*/*w*) H_2_O_2_ was pipetted into the cell, and spectrum acquisition was started immediately.

### 2.6. Statistics

The results of spectrophotometry measurements were presented in the form of the time dependencies of the solution absorbance at 405 nm (*A*_405_(*t*) curves). The *A*_405_(*t*) curves were compared based on the least square method. For each absorbance value at each time point, the standard deviation (*SD*) was taken to be equal to the root mean square (RMS) value and calculated as follows:

(3) $$SD=\sqrt{\frac{\sum_{i=1}^{n}{\left(A_{i}-\bar{A}\right)}^{2}}{n-1}},$$

where *n* is the number of technical replicates performed for each sample studied. In the spectrophotometry experiments, the number of technical replicates performed for each sample studied was no less than three.

## 3. Results

### 3.1. Atomic Force Microscopy

**Figure 3:**
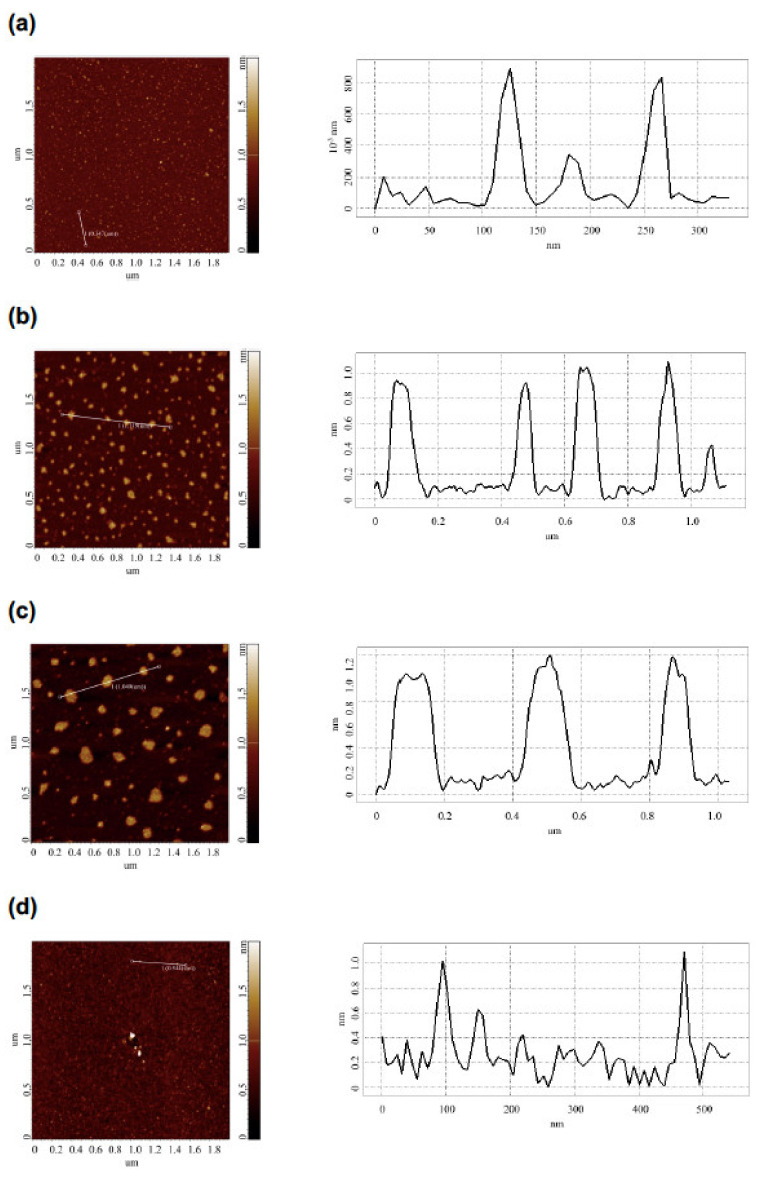
Typical AFM images (left panels) and respective cross-section profiles (corresponding to the lines in the AFM images; right panels) of mica-adsorbed HRP obtained with the enzyme solutions incubated at various distances *L* from the dodecahedron surface: *L* = 20 m (control experiment) (**a**); *L* = 0.03 m (**b**); *L* = 2 m (**c**); *L* = 5 m (**d**). Experimental conditions: HRP concentration 10^−7^ M in 2 mM PBSD buffer; incubation time 40 min. Scan size 2 × 2 µm, Z scale 2 nm.

The comparative AFM measurements of mica-adsorbed HRP were performed in order to investigate the effect of the incubation of the HRP enzyme solution at various distances from the dodecahedron on the enzyme’s adsorption behavior. The AFM measurements were performed in a tapping mode. Figure 3 displays the typical AFM images of the mica-adsorbed HRP obtained with the enzyme solutions incubated at the following distances from the dodecahedron surface:

1. Control experiment (*L*_1_ = 20 m);
2. Near the dodecahedron (*L*_2_ = 0.03 m);
3. At a middle distance (*L*_3_ = 2 m);
4. At a long distance (*L*_4_ = 5 m).

**Figure 4:**
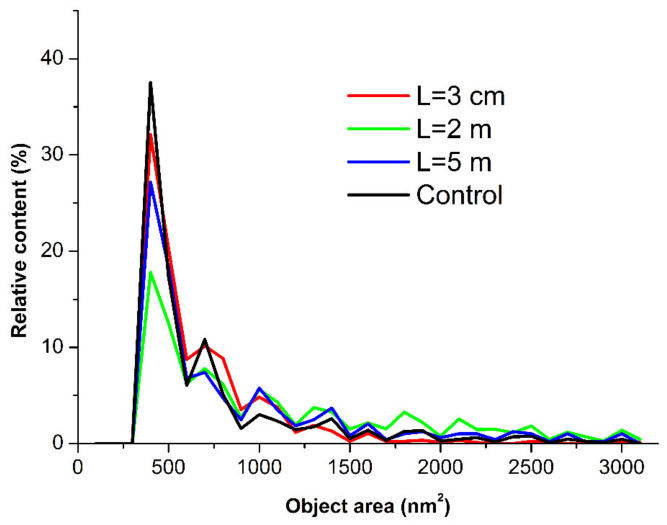
Relative distributions of the mica-adsorbed HRP particles with area *ρ*(*s*) obtained for the HRP samples incubated at various distances *L* from the dodecahedron: *L* = 0.03 m (red curve); *L* = 2 m (green curve); *L* = 5 m (blue curve); and *L* = 20 m (control experiment, black curve).

The AFM images shown in Figure 3 indicate that after incubation at any of the distances of *L* = 20 m (in control experiments), *L* = 5 m, or *L* = 0.03 m, the mica-adsorbed HRP was visualized as separate compact objects. At the same time, upon adsorption onto mica after the incubation at *L* = 2 m, HRP formed extended aggregate structures with lateral sizes of the order of 200 nm, which were much greater than in the case of the other distances (*L*). Figure 4 displays the relative distributions of the mica-adsorbed objects with the areas *ρ*(*s*) obtained for the HRP samples incubated at various distances *L*.

The *ρ*(*s*) curves shown in Figure 4 indicate that for the control sample incubated at *L* = 20 m, the maximum of the *ρ*(*s*) distribution was observed for the object area *s* = 400 nm^2^. The local maxima at 800, 1000, and 1400 nm^2^ were also clearly distinguished. For *s* > 1700 nm^2^, the *ρ*(*s*) values became comparable to the noise level.

At *L* = 0.03 m and *L* = 5 m, the absolute and local maxima of the respective *ρ*(*s*) distributions were observed at the same values of *s*, and the shape of the *ρ*(*s*) curves for these samples were similar to that obtained for the control sample. The *ρ*(*s*) values in these cases also became comparable to the noise level at *s* > 1700 nm^2^.

At *L* = 2 m, a decrease was observed in the content of the objects contributing to the absolute maximum of the respective *ρ*(*s*) distribution at *s* = 400 nm^2^, in comparison to the case with the control sample. At the same time, an increase was observed in the content of the objects contributing to the distribution’s right wing (with *s* ranging from 1700 to 2200 nm^2^).

**Figure 5:**
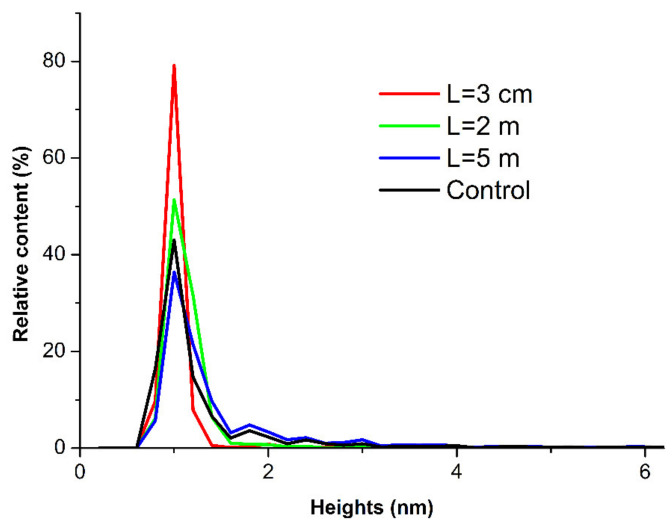
Relative distributions of the mica-adsorbed HRP particles with height *ρ*(*h*) obtained for the HRP samples incubated at various distances *L* from the dodecahedron: *L* = 0.03 m (red curve); *L* = 2 m (green curve); *L* = 5 m (blue curve); and *L* = 20 m (control experiment, black curve).

It is interesting to analyze the *ρ*(*h*) distributions obtained for the HRP samples studied. Figure 5 displays the *ρ*(*h*) curves obtained for the HRP samples incubated at various distances *L* from the dodecahedron.

The curves shown in Figure 5 indicate no significant differences between the *ρ*(*h*) distributions obtained for the samples incubated at various distances *L* from the dodecahedron. This means that, in comparison with the control sample, no change occurred in the *ρ*(*h*) distribution of the mica-adsorbed HRP after its incubation at any of the analyzed distances from the dodecahedron.

At the same time, the *ρ*(*s*) distribution obtained for the sample incubated at *L* = 2 m from the dodecahedron considerably differed from the *ρ*(*s*) distribution obtained for the control sample. This indicated an increased aggregation of HRP in the lateral direction upon its adsorption onto mica after the incubation of the enzyme solution at *L* = 2 m distance from the dodecahedron surface.

### 3.2. Spectrophotometry

**Figure 6:**
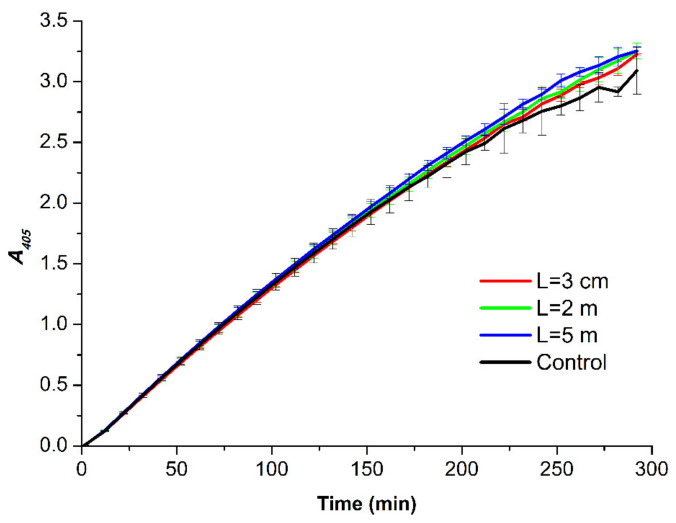
*A*_405_(*t*) curves obtained for the HRP samples incubated at various distances *L* from the dodecahedron: *L* = 0.03 m (red curve); *L* = 2 m (green curve); *L* = 5 m (blue curve); and *L* = 20 m (control experiment, black curve). Error bars indicate the SD values calculated according to Equation (1).

Figure 6 displays the time dependencies of the absorbance of the solutions containing HRP, incubated in the experimental setup, and its substrate ABTS at 405 nm wavelength (*A*_405_(*t*) curves).

A comparison of the experimental *A*_405_(*t*) curves shown in Figure 6 indicates that at each time point of the measurements, the difference in the absorbance recorded for the studied samples did not exceed the value of 2 × *SD*. Accordingly, no difference between the activity of the enzyme in the samples incubated in the experimental setup and that of the enzyme in the control sample was observed. This allowed us to conclude that under the conditions of our experiments, the incubation of the HRP solution near the dodecahedral structure did not affect the enzymatic activity.

## 4. Discussion

In the present study, the influence of the incubation of a solution of a model enzyme at various distances from a dodecahedral structure was studied using AFM and spectrophotometry. In order to better explain our experimental results, HRP enzyme glycoprotein was employed as a model object, since its properties are comprehensively described in the literature [41,49,50,51]. HRP is a 40 to 44 kDa [49,50] glycoprotein, with its structure comprising 13 α-helices and a short antiparallel β-sheet [52] stabilized by carbohydrate chains [50,51,53]. This enzyme forms aggregates in millimolar solutions [41]. It adsorbs onto bare mica as a mixture of monomeric and oligomeric (aggregated) forms [42,43,44,45]. Interestingly, HRP was found to serve as an indicator for the revelation of the effect of weak electromagnetic fields, and the action of such fields on HRP induces changes in its adsorption properties on mica [13,21,25].

In our experiments, we employed a well-practiced technique involving the direct adsorption of HRP particles from the sample solutions onto atomically smooth mica substrates with further visualization of the mica-adsorbed particles at the single-molecule level [13,21,25,42,43,44,45]. AFM is known to be an excellent tool for single-molecule studies [54,55]. Molecular absorption spectrophotometry has been used as a reference method, which has allowed for the estimation of whether the enzymatic activity of HRP is affected throughout the incubation near the dodecahedral structure [13,21,25,42,43,44,45].

Herein, the AFM-based technique was employed to determine whether the incubation of the HRP solution in a nonshielded laboratory room near a dodecahedral structure influences the properties of HRP. Electromagnetic radiation was not intentionally generated. It was found that the incubation near the dodecahedral structure influenced the adsorption behavior of HRP, while its enzymatic activity against the ABTS substrate remained unaffected. In addition, the effect of the incubation near the dodecahedron was nonlinearly dependent on the distance between the enzyme solution sample and the dodecahedron. In our experiments, this effect manifested itself only after the incubation of the HRP solution at a certain distance from the dodecahedron sample.

In our present work, the influence of the incubation of a buffered aqueous solution of a horseradish peroxidase enzyme near a structure with a dodecahedron shape on the enzyme properties was studied. The characteristic size of the dodecahedron’s edge was 0.23 m. The adsorption behavior of the HRP enzyme was investigated with the use of AFM, which allows one to visualize the objects formed by the HRP particles adsorbed onto the mica substrate during its incubation in the enzyme solution. This technique allowed us to obtain images of single enzyme molecules and their structures formed on the mica substrate surface. The AFM data obtained for the HRP solutions, incubated at various distances *L* from the dodecahedron surface, were analyzed, and the relative distributions of the mica-adsorbed objects with the area *ρ*(*s*) and height *ρ*(*h*) values were obtained for each *L* value studied. The effect of the incubation near the dodecahedron on the enzyme properties was found to be dependent on the distance *L* between the dodecahedron and the enzyme solution. The test tube with the enzyme solution was placed at any of the following distances *L* from the dodecahedron: (1) far away from the dodecahedron (*L* = 20 m, control experiment); (2) in the vicinity of the dodecahedron (*L* = 0.03 m); (3) *L* = 2 m; (4) *L* = 5 m.

After the incubation of the enzyme solution at either *L* = 0.03 m or *L* = 5 m, no effect on its adsorption behavior was revealed.

Interestingly, the incubation at *L* = 2 m led to the increased aggregation of the enzyme upon its adsorption onto mica, as compared with the control solution incubated at *L* = 20 m. In addition, the heights of the mica-adsorbed objects remained the same, and the aggregation of HRP on mica in the lateral direction was observed. The analysis of the *ρ*(*s*) distribution obtained after the incubation at *L* = 2 m revealed the formation of objects with areas *s* ranging from 1700 to 2200 nm^2^ along with objects with 400 nm^2^ corresponding to the maximum global distribution. The objects with a 400 nm^2^ area likely represent the broadened images of HRP monomers. The broadening of the AFM images was obviously caused by the influence of the AFM tip curvature radius, which was from 10 to 20 nm [39,56]. The local *ρ*(*s*) maxima at *s* = 800 nm^2^, *s* = 1000 nm^2^, and *s* = 1400 nm^2^, observed in the control experiments and after the incubation at *L* = 0.03 m and *L* = 5 m, corresponded to the aggregates of various orders.

The influence of the incubation of HRP at various distances from the dodecahedron on its enzymatic activity against ABTS was also studied. In comparison with the control solution, spectrophotometric measurements revealed no change in the activity of the enzyme against its substrate after its incubation at any of the distances studied. This confirms that the effect of the dodecahedral structure on HRP biomolecules is subtle and can only be revealed through AFM [25,57], as this effect is indistinguishable with the use of spectrophotometry.

Such a behavior of the enzyme indicates an absence of any considerable effect of the dodecahedron on the enzyme’s active site even in the case when its adsorption properties change (at *L* = 2 m). The change in the adsorption behavior of the enzyme observed after its incubation at *L* = 2 m can be connected with the alterations in the hydration of the surface groups of its globule [58], which participate in enzyme–enzyme and enzyme–mica surface interactions. In this context, these alterations occur away from its active site. It should be emphasized that the possibility of the occurrence of alterations in the hydration shell of biological macromolecules under the action of weak electromagnetic radiation was discussed by Pershin [58]. The influence of changes in the hydration of enzymes on their structure and activity was also reported in other papers. The hydration shell of a biomolecule is known to be an essential factor influencing biomolecular structure [59,60] and functioning [61], including enzymatic activity [61,62,63] and biomolecular recognition [64]. Regarding enzymes, water not only acts as a stabilizer of the enzyme conformation but also increases the flexibility of the enzyme macromolecule and the polarity of the active site [65].

The increased aggregation of the enzyme, incubated at *L* = 2 m, in the lateral direction can indicate an enhanced interaction between the enzyme globules, while the interaction between the enzyme and the substrate surface remains strong and does not allow the enzyme to form three-dimensional structures with greater heights during the aggregation. Such a dependence of the adsorption behavior of HRP on the distance from the dodecahedron can indicate a nonuniform spatial distribution of the electromagnetic field at the distances studied. The latter can occur owing to the reflection and refraction of the external background electromagnetic radiation from the dodecahedron surface. The change in the intensity of background electromagnetic radiation near objects of various shapes—for instance, near a pyramidal structure—was studied both theoretically [20] and experimentally [21,40]. The effect of spherical objects was also experimentally demonstrated [25]. These geometric bodies were shown to be able to redistribute the electromagnetic field topography, leading to an effect on the properties of an enzyme solution. In addition, the concentration of the electromagnetic field is known to occur in certain points of space with respect to the geometric body [20]. Specifically, by modeling the electromagnetic field distribution, Balezin et al. demonstrated that in the case of a pyramid, the electromagnetic field is concentrated at a characteristic distance from the pyramid’s base and at the distance of the order of the pyramid’s height [20]. A dodecahedron represents a body that can be inscribed into a sphere. At the same time, it has pentagonal faces. Accordingly, a dodecahedron can be presented as a system of pyramids, devoid of side faces but with a common center. With these considerations, a possibility of the influence of the entire dodecahedral structure on the enzyme solution, placed outside of this system of pyramids, was assumed. The characteristic length at which the concentration of the electromagnetic waves occurs was assumed to be proportional to the size of the dodecahedron. This size was ~0.64 m, and the effect on the enzyme solution was observed at a distance of about three dodecahedron sizes. This distance is probably the very one at which the external electromagnetic field topography considerably changed, influencing the enzyme solution.

It should be noted that even insignificant (near-background) changes in the electromagnetic field topology can considerably influence the enzyme’s properties. This was observed, for instance, in the case of knotted electromagnetic fields affecting the HRP enzyme [13]. It should be emphasized that, in our experiments reported herein, the electromagnetic field was not intentionally generated—as opposed to the case studied in [13].

## 5. Conclusions

The results of our study on the effect of a dodecahedron on an enzyme can be useful in the optimized construction of devices, the elements of which include dodecahedral structures (biosensors and other devices such as omnidirectional acoustic generators).

The influence of the incubation of a horseradish peroxidase solution at various distances from a dodecahedral structure on the enzyme’s properties was studied. The effect of such an incubation process was found to have a complex character dependent on the distance between the solution and the dodecahedron surface. An increased aggregation of the enzyme was observed upon its adsorption onto mica after the incubation of its solution at a 2 m distance from the dodecahedron surface. At longer (5 m and 20 m) and shorter (0.03 m) distances, no increase in the aggregation of HRP was observed.

The results obtained herein can be useful in the development of biosensors—particularly enzyme-based ones—and other electronic equipment, and in the development of models describing the action of electromagnetic radiation on biological systems.

## Data Availability

Correspondence and requests for materials should be addressed to Y.D.I.
